# Cumulative incidence of venous thromboembolism in patients with advanced cancer in prospective observational study

**DOI:** 10.1002/cam4.3670

**Published:** 2021-01-09

**Authors:** Hirotsugu Kenmotsu, Akifumi Notsu, Keita Mori, Shota Omori, Takahiro Tsushima, Yasuomi Satake, Yoshihiro Miki, Masakazu Abe, Masahito Ogiku, Toshio Nakamura, Masakazu Takagi, Hideto Ochiai, Hirofumi Yasui, Toshiaki Takahashi

**Affiliations:** ^1^ Division of Thoracic Oncology Shizuoka Cancer Center Shizuoka Japan; ^2^ Clinical Research Center Shizuoka Cancer Center Shizuoka Japan; ^3^ Division of Gastrointestinal Oncology Shizuoka Cancer Center Shizuoka Japan; ^4^ Department of Respiratory Medicine Shizuoka City Shizuoka Hospital Shizuoka Japan; ^5^ Department of Respiratory medicine Seirei Hamamatsu General Hospital Hamamatsu Japan; ^6^ Division of Gynecology Shizuoka Cancer Center Shizuoka Japan; ^7^ Gastroenterological Surgery Hamamatsu Medical Center Hamamatsu Japan; ^8^ Surgery Fujieda Municipal General Hospital Fujieda Japan; ^9^ Department of Gastroenterological surgery Shizuoka General Hospital Shizuoka Japan; ^10^ Department of Gastroenterological surgery Iwata City Hospital Iwata Japan

**Keywords:** advanced cancer, chemotherapy, deep vein thrombosis, pulmonary thromboembolism, venous thromboembolism

## Abstract

Venous thromboembolism (VTE) is frequently observed in patients with advanced cancer. The objective of this prospective observational study was to estimate, based on intensive screening, using computed tomography, lower‐extremity ultrasonography, and D‐dimer testing, the prevalence of VTE in patients with advanced cancer.

Patients with metastatic or locally advanced cancer without anticoagulant therapy, who were planning to receive chemotherapy during 4 weeks, were eligible. Evaluations of VTE were performed at pretreatment, 12 weeks, and 24 weeks after the start of chemotherapy. Primary endpoint was cumulative incidence of VTE for 24 weeks. Secondary endpoints included incidence of VTE (pretreatment, 12 weeks, and 24 weeks after the start of chemotherapy), VTE according to primary cancer site, symptomatic VTE, pulmonary thromboembolism (PE), and treatment of VTE.

We enrolled 860 patients with a median age of 68 years, including 34% female and 71% lung cancer. Cumulative incidence of VTE for 24 weeks was 22.6% (95% confidence interval: 19.8%–25.5%) (194 of 860 patients). Incidence of VTE was 11.3% pretreatment, 16.8% 12 weeks, and 14.1% 24 weeks. Symptomatic VTE was observed in 4.0% and PE in 1.0% of patients. By multivariate analysis, sex, D‐dimer level, and platelet count were independent risk factors of VTE for 24 weeks.

This large prospective observational study showed that cumulative incidence of VTE was high in advanced cancer patients, mainly lung cancer. Although most patients showed asymptomatic VTE, intensive screening of VTE may be considered in advanced cancer patients, especially in women with high level of D‐dimer and decreased platelet count (UMIN000015243).

## Graphical abstract

In 860 patients with advanced cancer, cumulative incidence of VTE for 24 weeks was 22.6% (95% confidence interval: 19.8%–25.5%). Incidence of VTE was 11.3% pretreatment, 16.8% 12 weeks, and 14.1% 24 weeks. By multivariate analysis, sex, D‐dimer level, and platelet count were independent risk factors of VTE for 24 weeks.

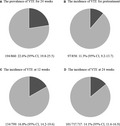

## 1 INTRODUCTION

Cancer is one of the most common risk factors for venous thromboembolism (VTE), including pulmonary embolism (PE) and deep vein thrombosis (DVT).^1^ The increased risk of VTE in cancer patients is greatest in the first few months after cancer diagnosis, and can persist for many years after an initial episode of symptomatic VTE.^1^ VTE is one of the most important causes of morbidity and mortality in cancer patients, and has a risk of worsening quality of life.^2^ In an epidemiological study of patients with or without cancer, Asians, compared with Caucasians, had 3–5‐fold lower incidence of symptomatic VTE.^3^ A large retrospective cohort study showed that Asians had a low risk of VTE by multivariate analysis.^4^ However, a Korean cohort study using prospective databases revealed that 3.5% of patients with gastric cancer had VTE.^5^ In a Japanese study, VTE was found in 4.8% of 272 patients with cervical cancer.^6^ Lower‐extremity ultrasonography is a standard imaging test to diagnose DVT, and enhanced chest computed tomography (CT) scan is that for PE. However, no large prospective study has evaluated the cumulative incidence of VTE based on intensive screening in patients with advanced cancer.

We conducted this prospective observational study (VISUAL study) to estimate the cumulative incidence of VTE in Japanese patients with advanced cancer, based on intensive screening, using enhanced chest CT scan, and lower‐extremity ultrasonography.

## 2 MATERIALS AND METHODS

### 2.1 Patients

Chemotherapy‐naïve adult patients aged ≥20 years with advanced or relapse cancer who planned to receive chemotherapy during 4 weeks, and Eastern Cooperative Oncology Group (ECOG) performance status 0–2, were eligible for this study. Patients with a diagnosis of VTE 4 weeks before enrollment, receiving anticoagulant therapy, or with known coagulation disorder were excluded.

### 2.2 Procedures

This prospective observational study was conducted at eight institutes in Shizuoka, Japan from December 2014 to June 2018. This study was conducted in accordance with the principles of the Declaration of Helsinki and the study protocol was approved by the Institutional Review Board of each study institution. All patients provided written informed consent prior to participation. This VISUAL study is registered at the University Hospital Medical Information Network (UMIN) Clinical Trial Registry (UMIN 000015243).

### 2.3 Endpoints

VTE was evaluated based on intensive screening, using contrast‐enhanced chest CT to evaluate the metastatic status and duplex ultrasonography of whole‐leg, pretreatment, 12 and 24 weeks after the start of chemotherapy. D‐dimer was tested pretreatment, 12 and 24 weeks after the start of chemotherapy. Diagnosis of VTE was performed by each physician, without central review. The primary endpoint, cumulative incidence of VTE (PE and DVT) for 24 weeks, was defined as the rate of VTE in all patients between the date of enrollment and 24 weeks later. Secondary endpoints included incidence of VTE at each point (pretreatment, 12 and 24 weeks after the start of chemotherapy); VTE for 24 weeks according to primary cancer site; symptomatic VTE and PE for 24 weeks; and treatment of VTE.

### 2.4 Statistical analyses

All patients initiating pretreatment evaluation were included in the statistical analysis. For sample size calculation, we assumed the population cumulative incidence of VTE was 15% and set a width of 5% for the 95% confidence interval (CI) of cumulative incidence. The corresponding sample size was 821. Finally, assuming dropout, the planned sample size was 1000. Although patient accrual was planned to be 2 years with follow‐up time of 6 months, enrollment was terminated at 3 years because of slow accrual. The full analysis set was defined as all enrolled patients excepting those with refusal and duplicate registration. The cumulative incidence of VTE (PE and DVT) for 24 weeks was evaluated using the full analysis set. We evaluated the incidence of VTE at each point for patients in the full analysis set who received VTE screening at each point. The cumulative incidence of VTE for 24 weeks according to primary cancer site, and symptomatic VTE and PE for 24 weeks were assessed in the full analysis set of patients who received VTE screening at least once. To identify predictive factors of VTE cumulative incidence for 24 weeks in patients with advanced solid cancer, univariate and multivariate logistic regression analyses were conducted. All *p* values were reported as two‐sided and *p* < 0.05 was considered statistically significant. 95% CIs were calculated using the Clopper‐Pearson method. We used R statistical package, version 3.5.1 (R Core Team, July 2018; www.r‐project.org) for statistical analysis.

## 3 RESULTS

### 3.1 Study population

**FIGURE 1:**
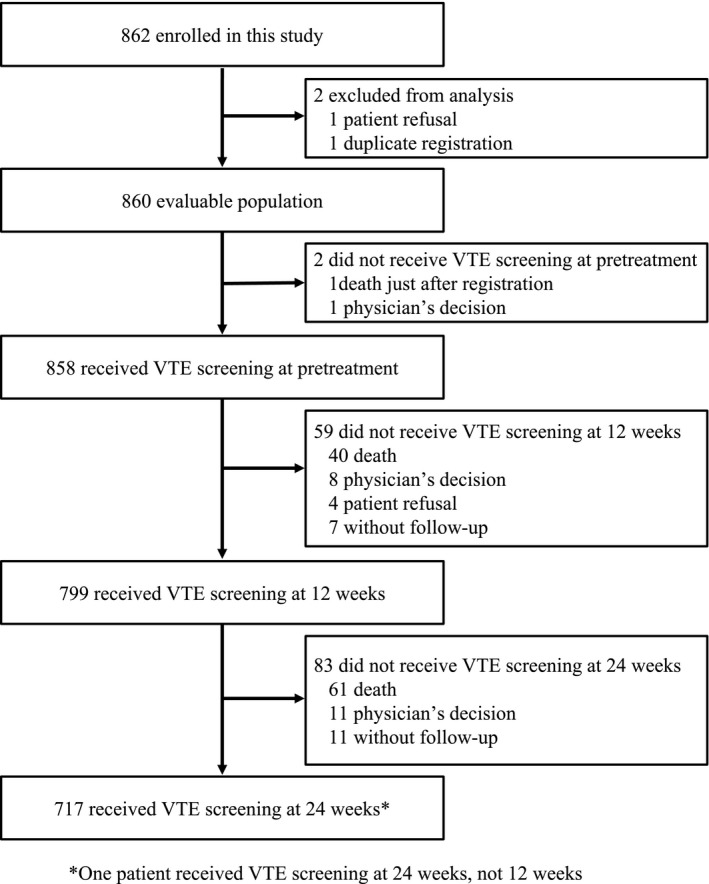
Patients screened for VTE pretreatment, 12 and 24 weeks after the start of chemotherapy. VTE, venous thromboembolism.

Between December 2014 and December 2017, we enrolled 862 patients. Two patients were excluded from the primary analysis; one for patient refusal and one with duplicate registration. There was no evaluation of VTE in two patients; one who died just after registration and one who did not receive any VTE screening (Figure 1). Table 1 summarizes the baseline characteristics of the enrolled patients. The median age was 68 years (range 28–96 years), 66% were male and 34% female, and 46%, 45%, and 8% had ECOG performance status 0, 1, and 2, respectively. Primary cancer site included lung (71%), gastrointestinal (GI) tract (15%), hepatobiliary and pancreatic system (5%), and gynecological tract (3%).

**TABLE 1:** Patient characteristics at baseline (*n *= 860)

	No. of patients	(%)
Sex		
Male	566	(66)
Female	294	(34)
Age, year		
Median	68	
(range)	(28‐96)	
Performance status (ECOG)		
0	394	(46)
1	391	(45)
2	72	(8)
3	3	(0)
Body mass index^a^, kg/m^2^		
Median	21.9	
(range)	(13.1‐40.9)	
Primary cancer site		
Lung	611	(71)
Gastrointestinal	125	(15)
Hepatobiliary and pancreatic	44	(5)
Gynecological	28	(3)
Breast	5	(1)
Urological	4	(0)
Head and Neck	2	(0)
Others	41	(5)
Disease status		
Locally advanced disease	188	(22)
Metastatic disease	672	(78)
Previously received surgery	182	(21)
Previously received radiotherapy	70	(8)
First‐line chemotherapy regimen		
Bevacizumab	68	(8)
Fluoropyrimidine	191	(22)
Taxanes	188	(22)
Platinum	611	(71)
Anti‐PD‐1 inhibitors	12	(1)
Tyrosine‐kinase inhibitors	132	(15)
D‐dimer^b^, μg/mL		
Median	1.2	
(range)	(0.1‐66.1)	
White blood cell^c^, /μL		
Median	6830	
(range)	(2300‐40340)	
Platelet^c^, /μL		
Median	249000	
(range)	(64000‐865000)	
Hemoglobin^c^, g/dL		
Median	13.1	
(range)	(7.2‐17.3)	

Abbreviation: ECOG: Eastern Cooperative Oncology Group.

^a^ Not evaluable in two patients.

^b^ not evaluable in 17 patients.

^c^ not evaluable in one patient.

### 3.2 VTE outcome

**FIGURE 2:**
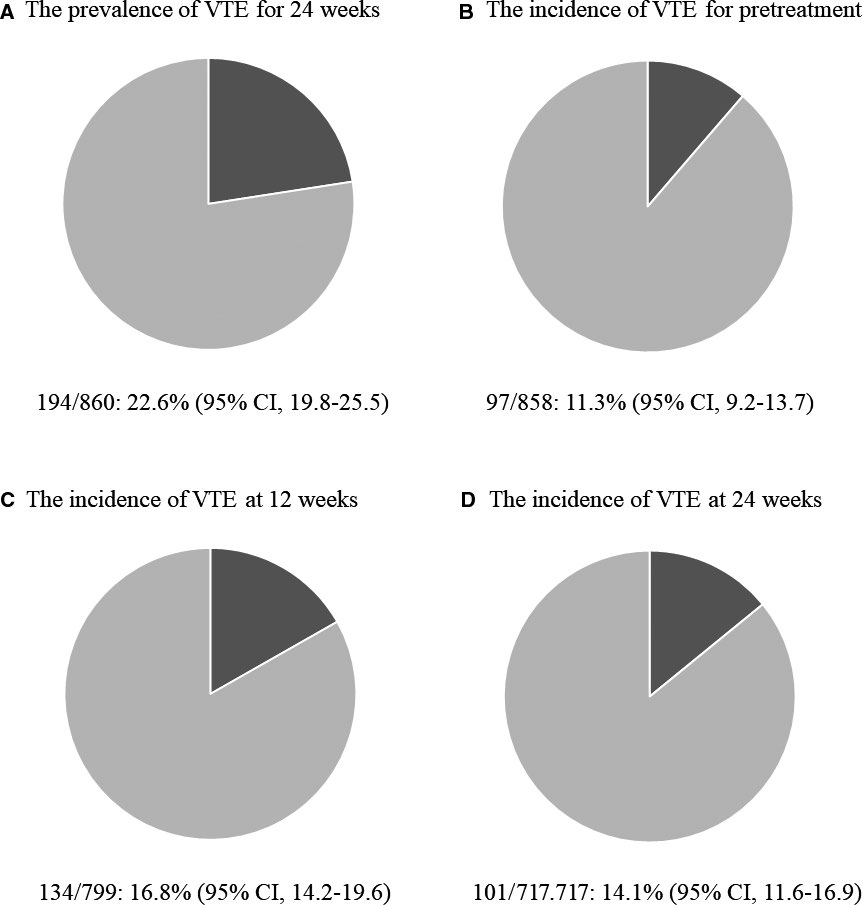
Cumulative incidence of VTE for 24 weeks, and incidence of VTE (pretreatment, 12 and 24 weeks after the start of chemotherapy). VTE, venous thromboembolism.

The primary endpoint, cumulative incidence of symptomatic and asymptomatic VTE (PE and DVT) for 24 weeks, was 22.6% (95% CI: 19.8%–25.5%) (194 of 860 patients). The incidence of VTE was 11.3% (95% CI: 9.2%–13.7%) (97 of 858 evaluable patients) pretreatment; 16.8% (95% CI: 14.2%–19.6%) (134 of 799 evaluable patients) at 12 weeks after the start of chemotherapy; and 14.1% (95% CI: 11.6%–16.9%) (101 of 717 evaluable patients) at 24 weeks after the start of chemotherapy (Figure 2). Symptomatic VTE for 24 weeks was observed in 4.0% (95% CI: 2.7%–5.5%) of 858 evaluable patients, and PE was observed in 1.0% (95% CI: 0.4%–2.0%). Table 2 summarizes the baseline characteristics of the patients with symptomatic and asymptomatic VTE for 24 weeks. The distribution of DVT was as follows: femoral (*n* = 12), popliteal (*n* = 18), and distal (*n* = 179). Among 23 patients showing symptomatic VTE at 12 or 24 weeks, 12 patients had a previous detection of asymptomatic VTE. Cumulative incidence of VTE for 24 weeks was 12.7% (95% CI: 10.4%–15.4%) of 761 patients who did not have VTE at pretreatment. Of the 194 patients with a diagnosis of VTE for 24 weeks after the start of chemotherapy, 129 (67.0%, 95% CI: 59.9%–73.6%) received anticoagulation therapies, including factor Xa inhibitors (*n* = 125), vitamin K antagonists (*n* = 7), and unfractionated heparin (*n* = 3). In 68 patients with VTE not receiving anticoagulation therapies, VTE was recovered later in 14 patients. One patient with asymptomatic VTE not receiving anticoagulation therapies showed symptomatic VTE. The cumulative incidence of VTE for 24 weeks was 24.1% in lung, 17.7% in GI, 25.6% in hepatobiliary and pancreatic, and 32.1% in gynecological cancer (Table 3).

**TABLE 2:** Patient characteristics in patient with VTE (n=194)

	Symptomatic VTE (*n *= 34)		Asymptomatic VTE (*n *= 160)	
	No. of patients	(%)	No. of patients	(%)
Sex				
Male	14	(41)	90	(56)
Female	20	(59)	70	(44)
Age, year				
Median	68.5		69.5	
(range)	(44‐86)		(38‐88)	
Performance status (ECOG)				
0	10	(29)	71	(44)
1	18	(53)	78	(49)
2	6	(18)	11	(7)
3	0	‐	0	‐
Body mass index, kg/m^2^				
Median	22.7		21.5	
(range)	(16.4‐37.1)		(14.5‐32.2)	
Primary cancer site				
Lung	20	(59)	127	(79)
Gastrointestinal	7	(21)	15	(9)
Hepatobiliary and pancreatic	4	(12)	7	(4)
Gynecological	2	(6)	7	(4)
Breast	0	‐	0	‐
Urological	0	‐	0	‐
Head and Neck	0	‐	0	‐
Others	1	(3)	4	(3)
Disease status				
Locally advanced disease	5	(15)	29	(18)
Metastatic disease	29	(85)	130	(81)
Previously received surgery	4	(12)	35	(22)
Previously received radiotherapy	1	(3)	15	(9)
First‐line chemotherapy regimen				
Bevacizumab	3	(9)	12	(8)
Fluoropyrimidine	8	(24)	27	(17)
Taxanes	6	(18)	29	(18)
Platinum	22	(65)	109	(68)
Anti‐PD‐1 inhibitors	0	‐	2	(1)
Tyrosine‐kinase inhibitors	8	(24)	28	(18)
D‐dimer^a^, μg/mL				
Median	3.7		2.0	
(range)	(0.6‐26.4)		(0.2‐66.1)	
White blood cell, /μL				
Median	7320		6880	
(range)	(3460‐13900)		(2300‐24230)	
Platelet, /μL				
Median	259000		236000	
(range)	(111000‐370000)		(64000‐511000)	
Hemoglobin, g/dL				
Median	12.7		13.0	
(range)	(7.3‐16.0)		(8.6‐17.1)	

Abbreviations: VTE, venous thromboembolism; ECOG, Eastern Cooperative Oncology Group.

^a^ Not evaluable in 5 patients.

**TABLE 3:** Cumulative incidence of VTE by primary cancer site (*n *= 858)

	No. of patients	VTE	(%)	95% confidence interval
Lung	611	147	24.1	20.7 – 27.7
Gastrointestinal	124	22	17.7	11.4 – 25.7
Hepatobiliary and pancreatic	43	11	25.6	13.5 – 41.2
Gynecological	28	9	32.1	15.8 – 52.4
Breast	5	0	0	0 – 52.2
Urological	4	0	0	0 – 60.3
Head and neck	2	0	0	0 – 84.2
Other	41	5	12.2	4 – 26.2

Abbreviation: VTE, venous thromboembolism.

### 3.3 Risk factors of VTE for 24 weeks

The results of the univariate analysis of risk factors for VTE for 24 weeks are shown in Table 4. Female sex, D‐dimer level ≥1.5 μg/mL, and platelet count <3,50,000/μL were significantly associated with incidence of VTE. Multivariate analyses were performed using three variables (sex, D‐dimer level, and platelet count), and demonstrated that female sex (odds ratio [OR]: 1.84, 95% CI: 1.30%–2.60%), D‐dimer level ≥1.5 μg/mL (OR: 2.81, 95% CI: 2.0%–3.97%), and platelet count <3,50,000/μL (OR: 2.85, 95% CI: 1.70%–5.01%) were significant independent risk factors of VTE for 24 weeks (Table 4). Even in patients excluding gynecological and breast cancers, multivariate analyses also demonstrated that female sex, D‐dimer level ≥1.5 μg/mL, and platelet count <3,50,000/μL were significant independent risk factors of VTE for 24 weeks (Table S1). In subgroup univariate analysis of patients with lung cancer, female sex and D‐dimer level ≥1.5 μg/mL were also significantly associated with cumulative incidence of VTE for 24 weeks. Receiving EGFR‐tyrosine kinase inhibitors and platelet count <3,50,000/μL were marginally associated with cumulative incidence of VTE. Multivariate analyses also showed that female sex (OR: 1.82, 95% CI: 1.19%–2.80%), D‐dimer level ≥1.5 μg/mL (OR: 2.54, 95% CI: 1.72%–3.78%), and platelet count (<3,50,000/μL) (OR: 2.17, 95% CI: 1.19%–4.23%) were significant independent risk factors of VTE in patients with lung cancer (Table 5).

**TABLE 4:** Univariate and multivariate analysis of VTE (*n *= 858)

	VTE (+)		VTE (‐)		Univariate		Multivariate	
		(%)		(%)	OR (95% CI)	*p*‐value	OR (95% CI)	*p*‐value
Sex								
Male	104	(18)	460	(82)	1		1	
Female	90	(31)	204	(69)	1.95 (1.41 – 2.71)	< 0.001	1.84 (1.30 – 2.60)	0.001
ECOG‐PS								
0	81	(21)	312	(79)	1			
1	96	(25)	294	(75)	1.26 (0.90 – 1.76)	0.181		
2‐3	17	(23)	58	(77)	1.13 (0.61 – 2.01)	0.689		
Body mass index								
<35 kg/m^2^	193	(23)	660	(77)	1			
≥35 kg/m^2^	1	(33)	2	(67)	1.71 (0.08 – 17.94)	0.662		
Surgery								
(‐)	155	(23)	522	(77)	1			
(+)	39	(22)	142	(78)	0.92 (0.62 – 1.36)	0.700		
Radiotherapy								
(‐)	178	(23)	610	(77)	1			
(+)	16	(23)	54	(77)	1.02 (0.55 – 1.78)	0.959		
Primary cancer								
Others	31	(17)	147	(83)	1			
Lung, Gynecologic	151	(24)	474	(76)	1.51 (1.00 – 2.35)	0.059		
Gastric, Pancreatic	12	(22)	43	(78)	1.32 (0.61 – 2.74)	0.463		
D‐dimer level								
<1.5 μg/mL	72	(15)	406	(85)	1			
≥1.5 μg/mL	117	(32)	247	(68)	2.67 (1.92 – 3.74)	< 0.001	2.81 (2.00 – 3.97)	< 0.001
White blood cell count								
≤11,000/μL	179	(23)	607	(77)	1			
>11,000/μL	15	(21)	57	(79)	0.89 (0.48 – 1.57)	0.707		
Platelet count								
≥3,50,000/μL	19	(14)	121	(86)	1			
<3,50,000/μL	175	(24)	543	(76)	2.05 (1.26 – 3.52)	0.006	2.85 (1.70 – 5.01)	< 0.001
Hemoglobin								
≥10 g/dL	182	(22)	634	(78)	1			
<10 g/dL	12	(29)	30	(71)	1.39 (0.67 – 2.71)	0.346		

Abbreviations: CI, confidence interval; ECOG‐PS, Eastern Cooperative Oncology Group performance status; OR, odds ratio; VTE, venous thromboembolism.

**TABLE 5:** Univariate and multivariate analysis of VTE in patients with lung cancer (n=611)

	VTE (+)		VTE (‐)		Univariate		Multivariate	
		(%)		(%)	OR (95% CI)	*p*‐value	OR (95% CI)	*p*‐value
Gender								
Male	85	(20)	337	(80)	1		1	
Female	62	(33)	127	(67)	1.94 (1.31 – 2.84)	0.001	1.82 (1.19 – 2.80)	0.006
ECOG‐PS								
0	52	(20)	206	(80)	1			
1	80	(27)	217	(73)	1.46 (0.98 – 2.18)	0.062		
2‐3	15	(27)	41	(73)	1.45 (0.73 – 2.77)	0.274		
Body mass index								
<35 kg/m^2^	146	(24)	462	(76)	1			
≥35 kg/m^2^	1	(100)	0		NA			
Surgery								
(‐)	125	(24)	387	(76)	1			
(+)	22	(22)	77	(78)	0.88 (0.52 – 1.46)	0.641		
Radiotherapy								
(‐)	133	(24)	417	(76)	1			
(+)	14	(23)	47	(77)	0.93 (0.48 – 1.71)	0.831		
EGFR‐TKI								
Not treated	113	(23)	387	(77)	1		1	
Treated	34	(31)	77	(69)	1.51 (0.95 – 2.37)	0.075	0.95 (0.56 – 1.57)	0.841
D‐dimer level								
<1.5 μg/mL	61	(17)	298	(83)	1		1	
≥1.5 μg/mL	82	(34)	161	(66)	2.49 (1.70 – 3.66)	< 0.001	2.54 (1.72 – 3.78)	< 0.001
White blood cell count								
≤11,000/μL	135	(24)	426	(76)	1			
>11,000/μL	12	(24)	38	(76)	1.00 (0.49 – 1.91)	0.992		
Platelet count								
≥3,50,000/μL	14	(16)	74	(84)	1		1	
<3,50,000/μL	133	(25)	390	(75)	1.80 (1.01 – 3.42)	0.056	2.17 (1.19 – 4.23)	0.016
Hemoglobin								
≥10 g/dL	141	(24)	451	(76)	1			
<10 g/dL	6	(32)	13	(68)	1.48 (0.51 – 3.81)	0.439		

Abbreviations: CI, confidence interval; ECOG‐PS, Eastern Cooperative Oncology Group performance status; EGFR‐TKI, EGFR tyrosine kinase inhibitors; OR, odds ratio.

## 4 DISCUSSION

This VISUAL study showed that compared with previous reports, cumulative incidence of VTE was relatively higher in Japanese patients with advanced cancer under intensive screening.^5^, ^7^, ^8^, ^9^ Although many previous studies have evaluated VTE in cancer patients, few have evaluated the incidence of VTE in cancer patients by both enhanced CT and lower‐extremity ultrasonography. Retrospective studies to evaluate the incidence of VTE in more than 1000 patients,^5^, ^7^, ^9^ and prospective studies did not have enough statistical power to evaluate cumulative incidence of VTE in cancer patients receiving chemotherapy.^10^, ^11^ To our knowledge, the present prospective study is the largest study to evaluate the incidence of VTE by both enhanced CT and lower‐extremity ultrasonography. The cumulative incidence of VTE for 24 weeks was 22.6%, which is similar to previous studies of Asian patients with cancer receiving chemotherapy.^10^, ^11^, ^12^ Although these prospective studies to evaluate incidence of VTE included 97–140 patients, the present VISUAL study registered 862 patients. Symptomatic VTE for 24 weeks was observed in 4.0% and PE in 1.0% of patients, which is similar to previous retrospective studies.^7^, ^9^

Among 761 patients without VTE at pretreatment, 12.7% had detection of VTE during chemotherapy for 24 weeks. The VISUAL study evaluated patients up to 24 weeks after the start of chemotherapy; thus, follow‐up time was longer than in previous prospective studies.^7^, ^10^, ^11^ Large randomized studies of efficacy of low‐molecular‐weight heparin (LMWH) for cancer patients receiving chemotherapy showed reduced incidence of thromboembolic events.^13^, ^14^ Recently, two phase III studies evaluating thromboprophylaxis with direct oral anticoagulants showed a reduction in VTE in high‐risk ambulatory patients with cancer.^15^, ^16^ A meta‐analysis showed that LMWH prophylaxis reduced the risk of VTE but did not significantly affect overall survival in patients with lung cancer.^17^ However, cumulative incidence of VTE was relatively higher in this study under intensive screening, and about 20% of patients with advanced cancer might had better to receive thromboprophylaxis. Routine pharmacological thromboprophylaxis is not recommended for outpatients with cancer, but may be considered in high‐risk ambulatory cancer patients.^18^, ^19^

The Khorana score (site of cancer, prechemotherapy platelet count, hemoglobin level, prechemotherapy leukocyte, and body mass index) is reported to identify cancer patients with high risk of VTE both at baseline and during treatment.^7^, ^20^ In a Korean prospective study of elderly cancer patients, female sex was reported to be a risk factor for VTE.^11^ Large retrospective studies have also shown that female sex is a risk factor for VTE in cancer patients.^4^, ^21^ In a recent study including two prospective cohorts, type of cancer and D‐dimer levels were risk factors for cancer‐associated VTE.^22^ A Japanese study showed that increased levels of D‐dimer were associated with risk of silent VTE in patients with ovarian cancer, and a suitable cut‐off value for detecting VTE seemed to be 1.5 μg/mL.^12^ Therefore, female sex and D‐dimer level were confirmed as risk factors for VTE in patients with cancer in this study. In a prospective observational study of patients initiating chemotherapy, elevated pretreatment platelet count was a significant risk factor for VTE.^7^ In contrast, the VISUAL study showed that decreased platelet count was a significant risk factor for VTE in Japanese patients with advanced cancer, mainly lung cancer. Although the reason for this difference is unclear, the previous study suggested that cumulative incidence of VTE varied among different ethnic groups. About 16% of patients showed platelet count ≥3,50,000/μL in this study, and cut‐off of platelet count might be not fully evaluated in Asian patients.

The VISUAL study had some limitations. First, this VISUAL study enrollment was terminated at 3 years because of slow accrual. Since dropout or ineligible patients were so few, the number of patients in the full analysis set who received VTE screening was considered to be enough to evaluate the cumulative incidence of VTE. Second, patients with lung (71%) and GI (15%) cancers were dominant; thus, there was imbalance among primary cancer sites. In this study, primary cancer site was not a significant risk factor in multivariate analysis. Since primary cancer site has been reported to be a significant risk of VTE, this study might have not enough power to detect risk by primary cancer site. Third, our study did not show data about hospitalization. In Japan, patients with advanced cancer have short hospitalization for induction of initial chemotherapy. Therefore, it was difficult to evaluate hospitalization. Fourth, since contrast‐enhanced chest CT was performed to evaluate metastatic status, not dynamic CT to evaluate PE, cumulative incidence of PE might be lower in this study. In addition, the accuracy of ultrasonography to evaluate DVT also sometimes vary in patients with asymptomatic DVT.^23^

In conclusion, this large prospective observational study showed that cumulative incidence of VTE was high under intensive screening, even in Japanese patients with advanced cancer, mainly lung cancer. Although most patients with VTE were asymptomatic, intensive screening of VTE may be considered in high‐risk ambulatory cancer patients, especially in women with high level of D‐dimer and decreased platelet count.

## FUNDING INFORMATION

We received funding from the Mt. Fuji Foundation for Healthcare Innovation and Cluster Development‐Pharma Valley Center.

## DISCLOSURE

Dr. Kenmotsu reports grants and personal fees from AstraZeneca K.K., grants and personal fees from Chugai Pharmaceutical Co, Ltd., personal fees from Ono Pharmaceutical Co, Ltd., and grants from Daiichi‐Sankyo Co., Ltd.

Dr. Takahashi reports grants and personal fees from AstraZeneca KK, grants and personal fees from Chugai PHARMACEUTICAL CO., LTD., grants and personal fees from Eli Lilly Japan K.K., grants from ONO PHARMACEUTICAL CO., LTD., grants from MSD K.K., and grants from Pfizer Japan Inc.

All remaining authors have declared no conflict of interest.

## Supporting information

Table S1

## Data Availability

The data sets used and/or analyzed during the current study are available from the corresponding author on reasonable request.
