# Supplementary material for: Cumulative incidence of venous thromboembolism in patients with advanced cancer in prospective observational study
Source: Cancer Med. 2021 Jan 9;10(3):895–904. doi: 10.1002/cam4.3670 (PMC7897954; doi:10.1002/cam4.3670)
Supplement: Supplementary file 1 — Table S1 [file CAM4-10-895-s001.docx]

**Table S1. Univariate and multivariate analysis of VTE in patients with advanced cancer, excluding** **gynecological and breast cancers (*n*=825)**

|  | VTE (+) | | VTE (-) | | Univariate | | Multivariate | |
| --- | --- | --- | --- | --- | --- | --- | --- | --- |
|  |  | (%) |  | (%) | OR (95%CI) | p-value | OR (95%CI) | p-value |
| Sex |  |  |  |  |  |  |  |  |
| Male | 104 | (18) | 459 | (82) | 1 |  | 1 |  |
| Female | 81 | (31) | 181 | (69) | 1.98 (1.41 – 2.77) | < 0.001 | 1.87 (1.31 – 2.68) | 0.001 |
| ECOG-PS |  |  |  |  |  |  |  |  |
| 0 | 77 | (20) | 302 | (80) | 1 |  |  |  |
| 1 | 92 | (24) | 285 | (76) | 1.27 (0.90 – 1.79) | 0.178 |  |  |
| 2-3 | 16 | (23) | 53 | (77) | 1.18 (0.63 – 2.14) | 0.589 |  |  |
| Body mass index |  |  |  |  |  |  |  |  |
| < 35 kg/m^2^ | 184 | (22) | 636 | (78) | 1 |  |  |  |
| ≥ 35 kg/m^2^ | 1 | (33) | 2 | (67) | 1.73 (0.08 – 18.14) | 0.656 |  |  |
| Surgery |  |  |  |  |  |  |  |  |
| (-) | 152 | (23) | 505 | (77) | 1 |  |  |  |
| (+) | 33 | (20) | 135 | (80) | 0.81 (0.53 – 1.23) | 0.333 |  |  |
| Radiotherapy |  |  |  |  |  |  |  |  |
| (-) | 170 | (22) | 586 | (78) | 1 |  |  |  |
| (+) | 15 | (22) | 54 | (78) | 0.96 (0.51 – 1.70) | 0.887 |  |  |
| Primary cancer |  |  |  |  |  |  |  |  |
| Others | 28 | (17) | 136 | (83) | 1 |  |  |  |
| Lung | 145 | (24) | 461 | (76) | 1.53 (0.99 – 2.43) | 0.063 |  |  |
| Gastric, Pancreatic | 12 | (22) | 43 | (78) | 1.36 (0.62 – 2.84) | 0.432 |  |  |
| D-dimer level |  |  |  |  |  |  |  |  |
| < 1.5 μg/mL | 70 | (15) | 400 | (85) | 1 |  |  |  |
| ≥ 1.5 μg/mL | 111 | (33) | 230 | (67) | 2.76 (1.97 – 3.89) | < 0.001 | 2.93 (2.07 – 4.17) | < 0.001 |
| White blood cell count |  |  |  |  |  |  |  |  |
| ≤ 11000 /μL | 171 | (23) | 586 | (77) | 1 |  |  |  |
| > 11000 /μL | 14 | (21) | 54 | (79) | 0.89 (0.46 – 1.59) | 0.705 |  |  |
| Platelet count |  |  |  |  |  |  |  |  |
| ≥ 350000 /μL | 16 | (13) | 110 | (87) | 1 |  |  |  |
| < 350000 /μL | 169 | (24) | 530 | (76) | 2.19 (1.30 – 3.94) | 0.005 | 2.98 (1.72 – 5.49) | < 0.001 |
| Hemoglobin |  |  |  |  |  |  |  |  |
| ≥ 10 g/dL | 173 | (22) | 614 | (78) | 1 |  |  |  |
| < 10 g/dL | 12 | (32) | 26 | (68) | 1.64 (0.78 – 3.25) | 0.170 |  |  |

CI: confidence interval; ECOG-PS: Eastern Cooperative Oncology Group performance status; OR: odds ratio; VTE: venous thromboembolism.
